# Effects of robot viscous forces on arm movements in chronic stroke survivors: a randomized crossover study

**DOI:** 10.1186/s12984-020-00782-3

**Published:** 2020-11-24

**Authors:** Yazan Abdel Majeed, Saria Awadalla, James L. Patton

**Affiliations:** 1 Richard and Loan Hill Bioengineering Department, University of Illinois at Chicago, Morgan St, 60607 Chicago, USA; 2 School of Public Health, University of Illinois at Chicago, Taylor St, 60612 Chicago, USA; 3 Shirley Ryan AbilityLab, Erie St, 60611 Chicago, USA

**Keywords:** Stroke, Reaching, Speed, Viscosity, Crossover

## Abstract

**Background:**

Our previous work showed that speed is linked to the ability to recover in chronic stroke survivors. Participants moving faster on the first day of a 3-week study had greater improvements on the Wolf Motor Function Test.

**Methods:**

We examined the effects of three candidate speed-modifying fields in a crossover design: negative viscosity, positive viscosity, and a “breakthrough” force that vanishes after speed exceeds an individualized threshold.

**Results:**

Negative viscosity resulted in a significant speed increase when it was on. No lasting after effects on movement speed were observed from any of these treatments, however, training with negative viscosity led to significant improvements in movement accuracy and smoothness.

**Conclusions:**

Our results suggest that negative viscosity could be used as a treatment to augment the training process while still allowing participants to make their own volitional motions in practice.

**Trial registration:**

This study was approved by the Institutional Review Boards at Northwestern University (STU00206579) and the University of Illinois at Chicago (2018-1251).

## Background

Stroke neurorehabilitation often uses the unique aspects of technology to improve motor recovery. While some researchers endeavored to simply assist movement to more closely resemble healthy patterns [1–3], others have attempted to exploit unique capabilities of robotics or graphic feedback to encourage neuroplasticity by augmenting error [4–8]. Even some traditional physical therapy exercises use mirrors to get the paretic side of the body to imitate the non-paretic side [9]. These are beneficial but far from a complete cure, and it remains to be seen what strategies emerge as optimal and what might still be left undiscovered.

An alternative strategy is to first uncover the attributes associated with better clinical movement outcomes, and then target training around these [10, 11]. Our previous work [12] employed a data-driven approach to model participant improvement using metrics derived from the movements themselves. We found that participant movement speed during the initial evaluation was most predictive of clinical changes. This speed was also the most strongly correlated with changes in the Wolf Motor Function Test (WMFT), making heightened speed a possible intervention for stroke. However, before such an intervention might be tested in clinical trials, we need to establish effective methods for speeding up participants.

There are multiple possible training conditions that may achieve this increase, and here we compare three candidate classes of conditions. One approach to affect movement speed is to directly increase it with a negative viscous field; previous work [13–16] showed that training with negative viscosity can improve participant movement and movement generalization abilities. Another possibility is to leverage the motor control mechanisms of error augmentation and after effects. Under this paradigm, participants would train with positive viscosity, under the expectation that their speed would increase as an aftereffect of that training when these resistive forces are removed [6, 17]. Finally, some research has shown that combining a resistive paradigm with a reward mechanism [18] may help participants learn better. In this case, participants will move in a positive viscosity field that attempts to slow them down, but moving above a certain speed is rewarded by a “breakthrough” where resistance vanishes. Participants may bias movements towards higher speeds to avoid the resistance.

Though it is somewhat understandable why these training conditions would change participant movement speed, the change being an increase is less obvious, especially when it comes to positive viscosity and breakthrough. Many research studies have demonstrated that training under certain conditions that alter normal movement - or perception of it - will induce an aftereffect in the opposite direction. This was shown to be true for primates [19] and humans [20]. Our reasoning for including positive viscosity is that the aftereffect of slowing movement speed would be an increase in speed when the slowing forces are removed. The breakthrough condition leverages the idea of limit-push. There is some evidence that introducing a “penalty” for participant movements that are undesired, and removing that penalty when participants conform to desired movements will bias subsequent motion towards these desired patterns, this was demonstrated using robotic forces [21] and purely visual distortions [22]. By penalizing slower movements in our breakthrough condition, and rewarding faster movements with removal of that penalty, we hope to bias participant motion towards these higher speeds.

In this preliminary clinical study, we simply compared the effects of these three paradigms on participant speed. Our modest goal was to determine if it was possible to influence participants’ speed. If speed *is* something that can be changed, we will explore the more difficult question of its influence on functional recovery for a later trial. Chronic stroke survivors participated in a single-visit crossover trial, where they trained for a short time under these three conditions. While we were mainly interested in the direct- and after-effects of these force paradigms on participant movement speed, we examined their effects on other movement metrics as well, such as error, efficiency, and smoothness.

## Methods

### Participant population

**Fig. 1:**
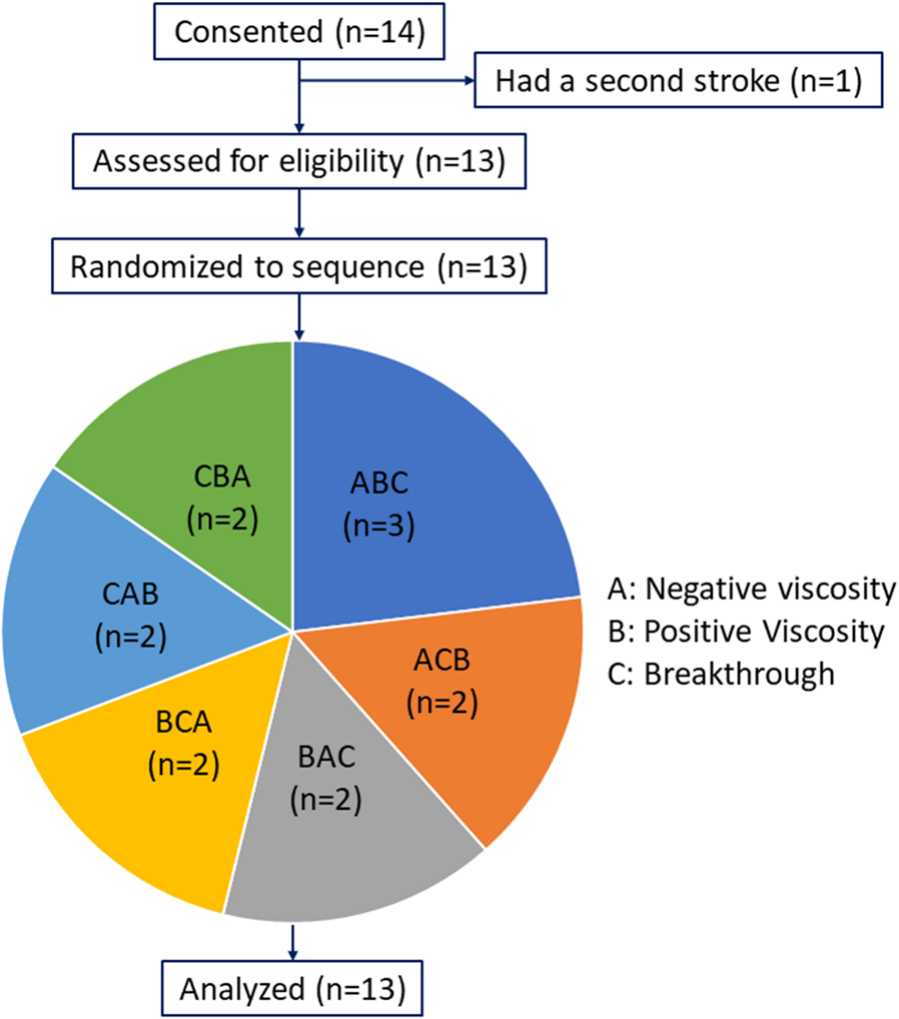
CONSORT-style diagram for the crossover study. The sequences participants were randomized to are shown in the pie-style diagram

We recruited 14 chronic hemiparetic stroke survivors (one participant was not able to take part in the study after consenting due to a second stroke), 15-50 on the Upper Extremity Fugl-Meyer scale. Participants were excluded if they had multiple lesions or multiple stroke events, if they had bilateral paresis, or if they had Botox^®^ treatments to the upper limbs within the last six months. Stroke survivors were recruited through the Clinical Neuroscience Research Registry of the Shirley Ryan AbilityLab and provided informed consent. This study was approved by the Institutional Review Boards at Northwestern University (STU00206579) and the University of Illinois at Chicago (2018-1251), and follows the guidelines of the Declaration of Helsinki. Figure 1 shows a CONSORT-style diagram (**CON**solidated **S**tandards Of **R**eporting **T**rials) for our crossover study, and Table 1 shows participant information at the time of participation in the study.

**Table 1:** Participant information for this study ($N = 14$)

Participant ID	Age (years)	Time since stroke (months)	Sex	Dominant side	Affected side	UEFM score
SP01	59	29	M	Right	Right	26
SP02	52	70	M	Right	Left	44
$\hbox {SP03}^{\mathrm{a}}$	64	29	M	Right	Right	39
SP04	59	45	M	Left	Right	37
SP05	37	52	M	Right	Right	32
SP06	64	95	M	Right	Left	35
SP07	83	32	M	Right	Right	46
SP08	45	75	F	Right	Left	23
SP09	67	11	M	Right	Left	37
SP10	50	157	F	Right	Left	33
SP11	58	50	F	Right	Right	40
SP12	57	29	F	Right	Left	24
SP13	67	49	M	Right	Right	23
SP14	46	43	F	Right	Left	34

$^{\mathrm{a}}$ SP03 consented but did not perform the study due to a second stroke

### Experiment design

**Fig. 2:**
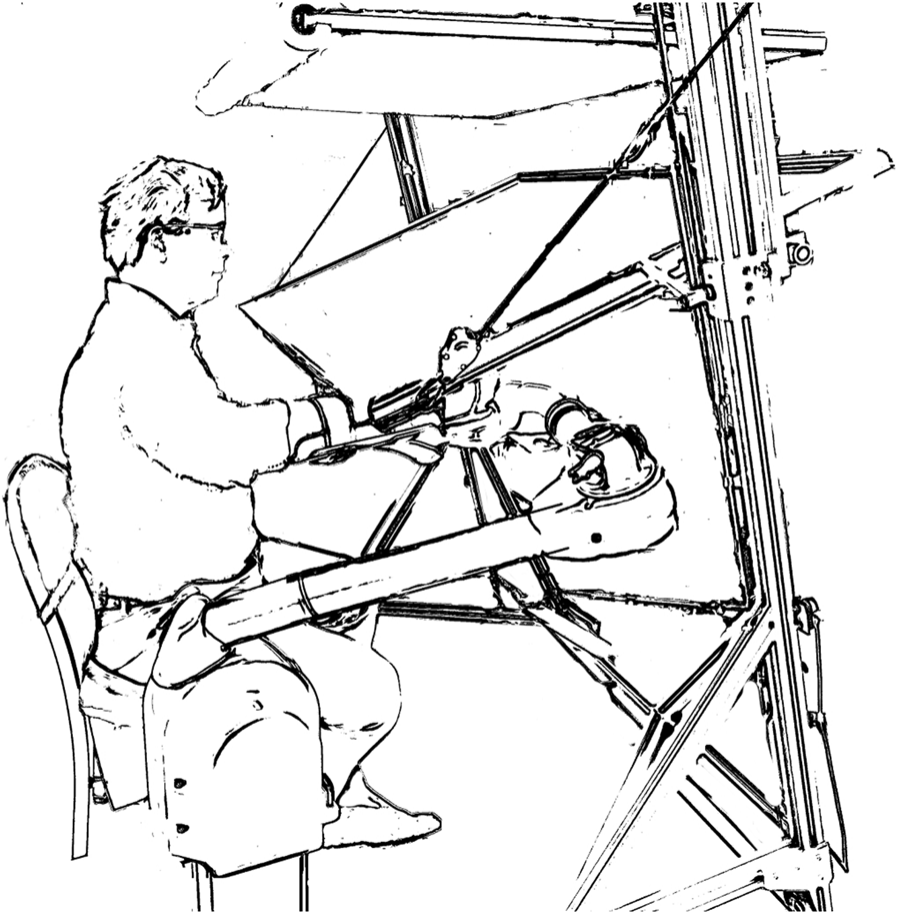
Experiment setup. Participants reached unimanually, alternating between a central home position and randomly to one of eight different target locations. The study was conducted using the Looking Glass virtual reality system and the Barrett Proficio robot arm

Participants completed a single-visit crossover study. Participants performed a targeted reaching task with their paretic arm attached to the Proficio^®^ 3-DoF robot from Barrett Technologies (Fig. 2). The Proficio allows three dimensional movement in a large workspace, approximating the normal range of human motion.

Participants started from a neutral home position that was customized for each person. Each trial consisted of a center-out motion to a spherical target with 3 cm diameter, 15 cm away. One of eight possible targets was displayed at random for each center-out reach. Participants then returned to the home position and the next target was displayed. Participants had 10 s to complete each trial, after which the trial ends, and the participants return to the home position.

**Fig. 3:**
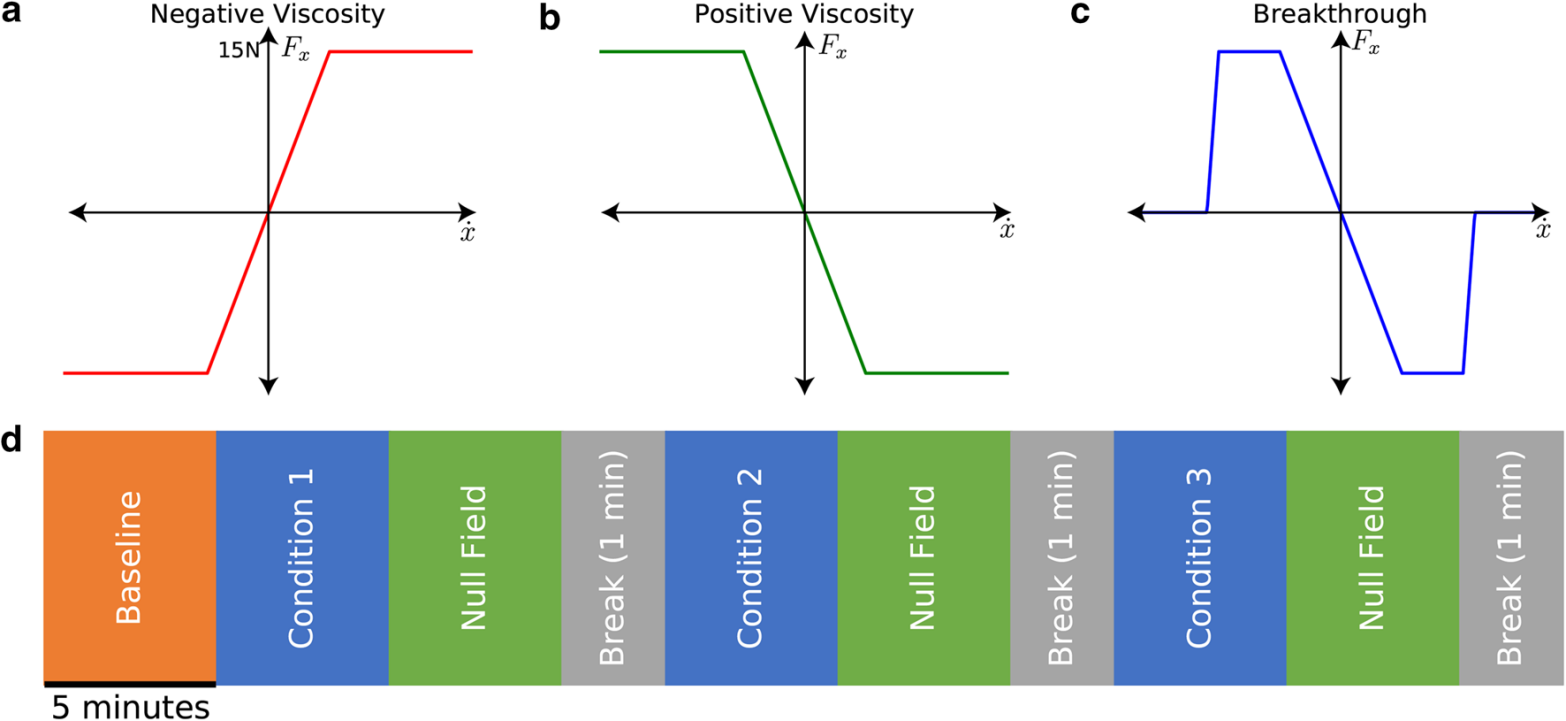
Experiment conditions and timeline **a** Timeline of the experiment, each “block” of trials lasted for 5 min, and there was a short break (30–60 s) in after each block. **b** Negative viscosity. Forces were proportional to velocity in all three directions, we drove the forces to zero at higher speeds for safety. **c** Positive viscosity. Similar to negative viscosity but the forces acted opposite to the direction of motion, slowing the participants. **d** Breakthrough. Forces were proportional to velocity until the participants reached 75% of their baseline speed, forces were then removed as a reward for reaching faster speeds

First, we evaluated baseline performance. Participants reached for five minutes to targets distributed in a quarter-sphere under no robotic forces. Participants then alternated between five minutes of reaching under each of the three experimental conditions, and five minutes of reaching with no forces to evaluate the aftereffects. The parts of the trial involving reaching under robotic forces did not include any “catch” trials where participants did not receive forces. This block design provided consistent rest periods, and consistent chunks of data for statistics. It also allows sufficient time for the effects of each force type to wash out before the next forces were presented. We presented the three conditions to each participant in a pseudo-random order. “Pseudo-random” refers to randomizing what each participant experienced while ensuring that we had at least 2 participant experiencing each of the 6 possible force orders, to control for any possible ordering effect. A simplified 1-D representation of the three force types for our experimental conditions is shown in Fig. 3a–c and the timeline of the experiment is shown in Fig. 3d.

### Data analysis

We resampled participant position data (originally at 1kHz) to 100Hz, then filtered it using a 7th order Butterworth filter with a 9Hz cutoff frequency. We extracted the following metrics from each movement, a lot of which are also explained in our previous paper [12]:

- *Movement accuracy (max perpendicular distance):* Participants were instructed to reach to the targets in a straight line, our chosen error measure was the maximum distance from the straight line between the initial position when the target was shown to the position of the target.
- *Max and mean speed:* We extracted the maximum speed during each trial, and calculated the mean speed as total path length divided by trial time.
- *Initial direction error:* We calculated the initial direction error as the angle (in radians) between the straight line to the target and the line connecting the initial position to the position where the participant reached 10% of their maximum speed. This was calculated independently for each trial.
- *Pre-movement speed:* We defined pre-movement speed as the average speed before participants ’launched’ their movement, defined as reaching 10% of their maximum speed.
- *Initial movement ratio:* Ratio of the distance covered during the first part of the movement (defined as the first speed peak, before making corrections) and the total movement distance.
- *Movement efficiency (path length ratio):* Ratio of the total movement distance and the straight line distance between the initial position and the target.
- *Movement smoothness (number of submovements):* We approximated the number of submovements as the number of speed peaks during each trial.
- *Speed ratio:* Ratio of the participant’s launch speed (speed of the first peak) and the maximum speed during the trial.
- *Percentage of movement in the target direction:* We calculated this metric by projecting the position vector between each pair of consecutive time samples onto the straight line from start to target, summing up the projections, and finally dividing by the hand path length.
- *Arrest period ratio:* The percentage of time during a trial where the participant was moving slower than 10% of max trial speed.

We focused our analysis for each condition (robot viscous force type) on groups of five trials: prior to exposure to the robotic forces (pre-exposure/baseline), when the forces were initially experienced (early exposure), at the end of the five minute block experiencing the forces (late exposure), the reaction to the forces being turned off (aftereffects), and the end of the null field block before the next condition is experienced (retention). We modeled study outcomes using linear mixed effects regression of the fixed conditions (vs baseline), time, and random subject effects.

$$\begin{aligned} y=x*\beta + Z * u + \epsilon \end{aligned}$$

where *y* represents the metric we are exploring, for example maximum speed, *X* represents the fixed effects (force condition, trial number), and *Z* represents the random effects (participant ID). We also made sure to check whether there was an interaction between the two fixed effects had an influence on the outcome, or whether the order of presenting the force conditions had an effect.

**Fig. 4:**
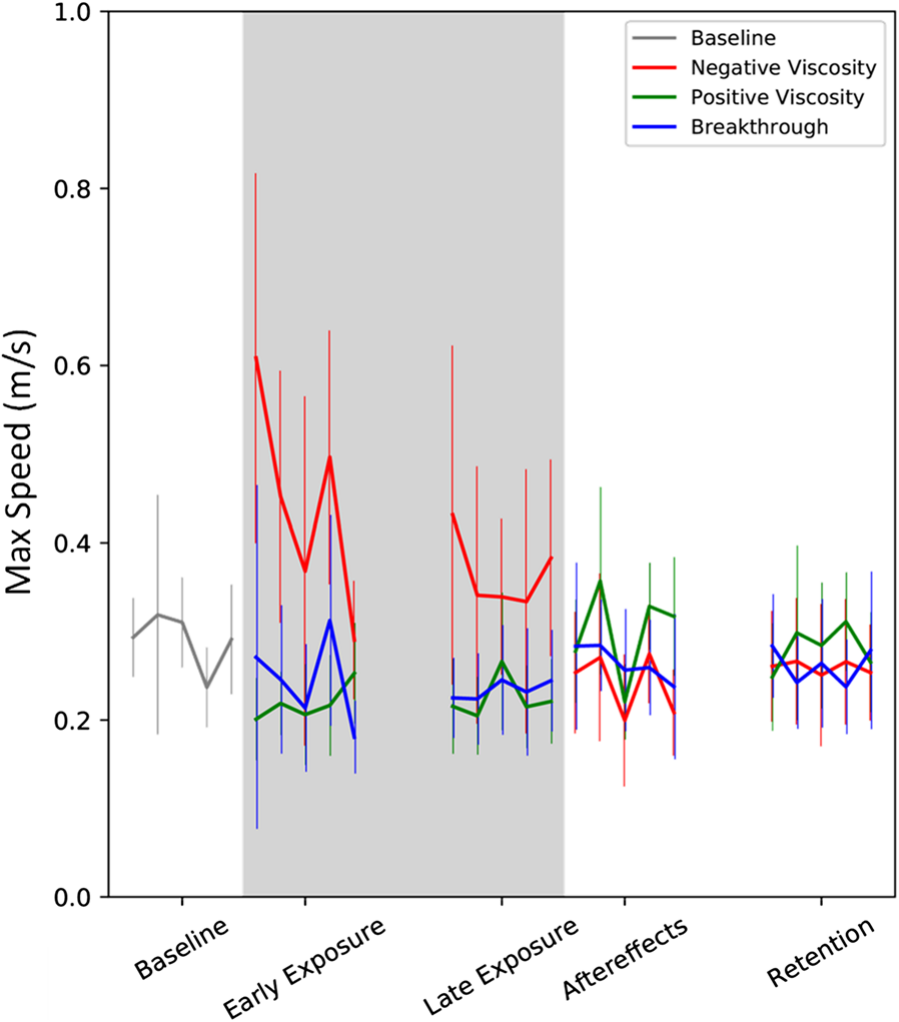
Effect of experiment conditions on altering participant speed. Thick lines represent means. Error bars represent $95\%$ confidence intervals across all participants. Each point on the thick lines is a trial. Robot forces were turned on in the shaded region

**Fig. 5:**
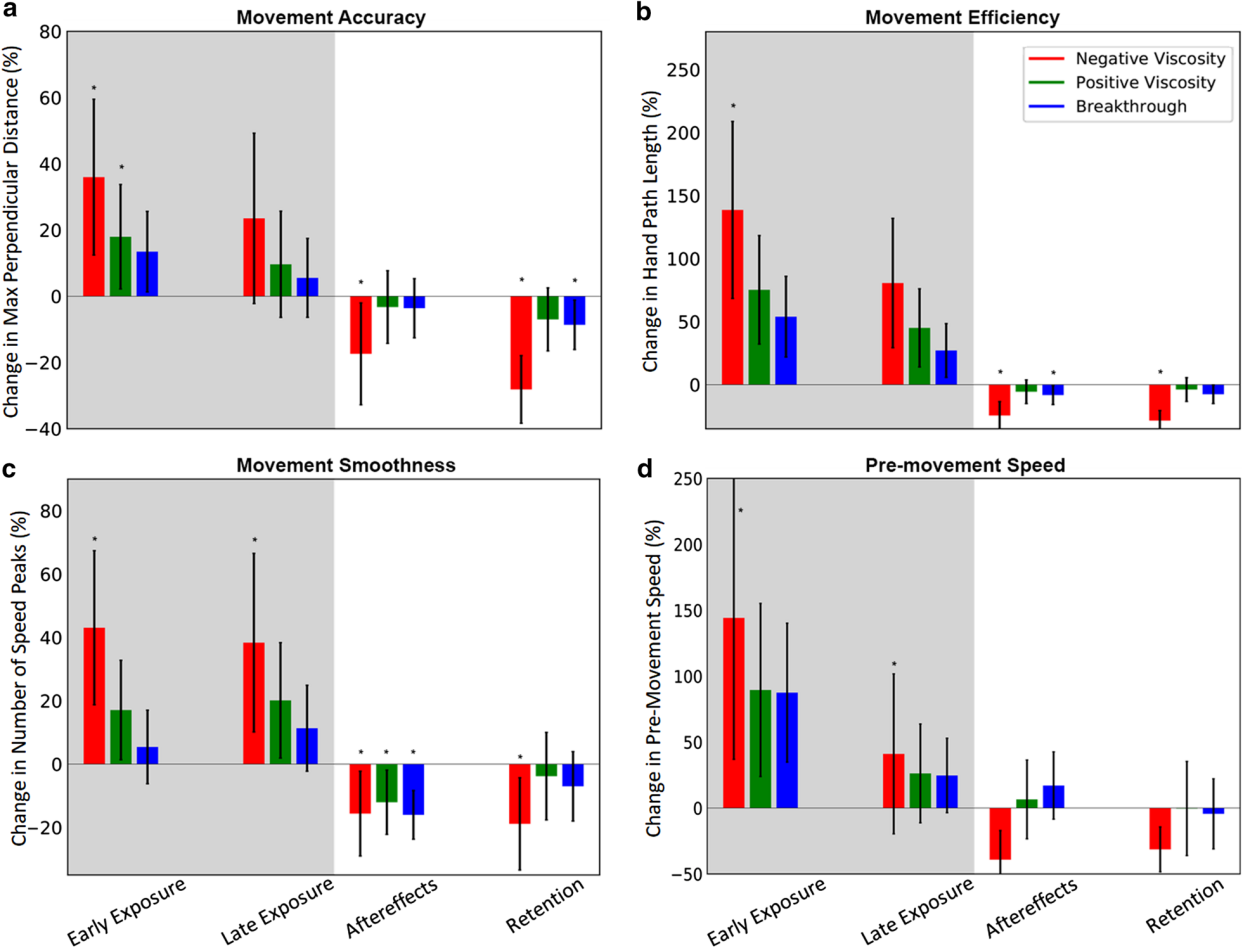
Effect of experiment conditions on various movement features. **a** Movement accuracy. **b** Movement efficiency. **c** Movement smoothness. **d** Pre-movement speed. Participants experienced robot forces in the shaded regions. The zero line represents the average of each metric during the baseline block, independently for each participant

We compared differences between condition pairs using post-hoc contrasts with Tukey’s adjustment for multiple comparisons in all cases. This was done regardless of whether the initial p-values were significant. We did not assume data normality in our mixed effects model or our statistical tests, however, since we had $>250$ trials in each phase of the study, we are leveraging the central limit theory and showing means and standard deviations in Figs. 4 and 5.

## Results

### Recruitement and participant issues

We enrolled one additional participant in case parts of the movement data from the first participant was unusable. Due to a participant initially having difficulty stopping their motion due to weakness in counteracting the negative viscosity forces. We were ultimately able to utilize the data we collected from all participants, and had a grand total of 13 participants.

### Movement speed

Negative viscosity produced significant speed increases during early exposure that persisted through late exposure ($p<0.0004$, Fig. 4). There was a significant difference between the effect of negative and positive viscosity forces ($p<0.001$), and between negative viscosity and breakthrough forces ($p<0.03$) during both early and late exposure. The order with which the force conditions were presented to the participants did not have a significant effect on speed ($p>0.16$). The aftereffect of negative viscosity was a significant decrease in speed ($p<0.02$) which did not persist at the end of washout (retention $p>0.09$).

During early exposure, neither breakthrough nor positive viscosity had any significant effects on movement speed ($p>0.11$). Both had a significant slowing effect on maximum speed ($p<0.03$) but not on mean speed ($p>0.055$) during late exposure. Neither had any significant effect on speed during washout ($p>0.07$). There was no significant difference in the effects of positive viscosity and breakthrough forces during any stage of the experiment ($p>0.48$). The effects of negative and positive viscosity forces were significantly different ($p<0.02$) for all but the retention stage ($p>0.61$).

### Other movement metrics

Movement error increased significantly during early exposure to negative viscosity ($p=0.004$, Fig. 5A). The effect was no longer significant by late exposure ($p=0.064$). However, both the aftereffect and retention had a significant reduction in movement error ($p<0.023$) compared to the baseline phase. Training with positive viscosity forces yielded a significant increase in movement error during exposure ($p<0.01$) but no significant aftereffects. Breakthrough forces had no significant effect on error during exposure, but showed a significant error reduction as an aftereffect ($p<0.02$). Interestingly, the retention movement error was affected by the order the force types were presented to participants ($p=0.04$), other stages were not affected by the order ($p>0.06$).

Movement distance showed behavior similar to movement error (Fig. 5b), where negative viscosity resulted in worse performance during exposure ($p<0.0001$) but significantly improved performance as an after effect ($p\le 0.001$). Positive viscosity had no significant effects during any stage, while breakthrough showed a significant improvement only during the aftereffect ($p\le 0.001$).

Movement smoothness, as quantified by the number of speed peaks, was significantly worse during exposure to negative viscosity ($p\le 0.0099$) and significantly better during washout ($p\le 0.033$, Fig. 5c) compared to baseline. Interestingly, all force types showed a significant improvement in movement smoothness in early aftereffects ($p\le 0.031$), though only after training with negative viscosity did participants retain that improvement.

Finally, there was a significant increase in pre-movement speed during exposure to negative viscosity (Fig. 5d). There was no significant effect for any of the three force types on this pre-movement speed by late washout, though training with negative viscosity showed an average reduction in pre-movement speed.

There were other smaller, mostly non-significant effects of the other force conditions on these movement metrics. Overall, participants did not improve their reaches after training with positive viscosity or breakthrough forces.

## Discussion

We looked for evidence of speeding participants up in both the direct effects when we turn on robotic forces and after effects after minutes of exposure then turning the forces off. As we expected, negative viscosity produced the largest direct effect (an increase in speed), since it was directly pushing participants to move faster. Speeds decreased by the end of exposure as they adapted, although they remained faster than baseline – a prolonged direct effect. Interestingly, we observed no speed-related after effects from any of our force treatments and any mismatch from baseline behaviour quickly dissipated.

While participants were reaching under the positive viscosity condition, they exhibited slower movements as we expected, though this slowdown was not statistically significant in most cases. There was a minor increase in movement speed as an aftereffect, which was also not significant. Ultimately, participants behaved as we expected during and after experiencing positive viscosity, but the effects were very small compared to their baseline movements.

We expected that breakthrough forces would bias towards faster movements during late exposure and the early aftereffect due to this condition rewarding faster movements. We instead saw a reduction in movement speed. This can be due to incomplete learning of the forces, where participants did not have enough experience with the breakthrough condition to sufficiently bias their movements. Another explanation can be that breakthrough forces do not have the desired effect on participant movement speed. Either way, in a direct comparison with negative and positive viscosity under similar force magnitudes and length of exposure, breakthrough forces did not succeed in increasing participant speed.

Negative viscosity reduces participants’ ability to control their arm movements during the initial ballistic phase. This led to a significant reduction in movement accuracy, effectiveness, and smoothness, as participants tried to counteract the destabilising force. These effects were reversed, as expected, once the forces were turned off, and participants significantly improved their reaches.

Although our long-term goals are to provide better rehabilitation (transferring to recovery and functional improvement), the goals of this paper were more modest, to determine if it is possible to influence participant speed. The goals of testing this in a protracted intervention will be tested in a later study. It remains to be seen whether increased speed from our work also has an effect.

We expected incomplete learning and/or incomplete washout due to our short exposure and null field blocks. Hence, we presented the forces to our participants in a randomized order and tested for any ordering effect. Out of 30 linear mixed effects models, the order the forces were experienced was significant in only two cases: late exposure movement distance, and retention movement error. This could be due to a few outliers in our small sample of participants, since in both cases the p-values were just below the significance level ($p\approx 0.04$).

Our goal with a crossover design was to elucidate the effects three robotic forces with heterogeneous stroke subjects, but there are intrinsic limitations with a crossover study. First, there may be an order (carryover) effect that can obscure the effects of any one treatment. In randomizing the order into all possible presentations (6 groups), we did our best at dealing with this. With $3! = 6$ possible order sequences for the three force types, we wanted to examine data from at least two participants per order sequence (1). Another limitation of crossover designs is the reduction in statistical power. This pilot study was not prospectively powered for significant outcomes due to lack of preliminary data. In spite of this, our work provided significant positive outcomes for some of the conditions (negative viscosity), which should guide future studies that employ this type of speed interventions. A third limitation is increased subject dropout, which was luckily not a problem in our case.

It is paradoxical that a speed-dependent force field that enhances velocities might also exacerbate spasticity or rigidity of the arm. Spasticity is defined as a hyper-excitable velocity-dependent stretch reflex [23, 24] and is typically measured with the modified Ashworth scale (MAS). Other researchers have shown that progressive reflex increases with repetitive stimuli (i.e., “windup”) in patients [25]. These repetitive stimuli may have different effects on the arm’s spasticity response depending on where the patient falls on the MAS. We happened to only get participants who did not have significant contractures or an MAS score of more than 3, as assessed by an occupational therapist. Modified Ashworth, the measure of spasticity, was not controlled for in this study, so it remains to be seen whether spastic participants are more or less likely to respond to our speed-enhancement in training.

Since we were conditioning the arm, repeated exposure to a stimulus that causes spastic responses may have a therapeutic effect. Training with velocity-enhancing fields showed that negative viscosity can improve participant movement and movement generalization abilities [13–16]. Our work here also did not definitively address the responsiveness to prolonged speed training, where spastic post-stroke patients may respond differently to such therapy. Interestingly, Park, et al. (NNR 2016) [26] also showed that training that emphasizes fast movements (therefore high speeds) leads to large improvements, which bolsters the hypothesis that training with negative viscosity may improve patient performance. Their (unassisted) training method led to significant fatigue [27]. We believe that fatigue is not an issue with our paradigm, since the patients do not have to support the weight of their arm against gravity. The robot performs gravity compensation thereby significantly reducing fatigue while performing the reaching task.

The rehabilitation literature supports that a more demanding training protocol leads to faster learning and improvements [28–31]. This study was impossible to control for this, as speed is part of any energetic calculation [32], but difference in metabolic cost across conditions may also influence outcome. It may be, that although speed was a target and proved to be influenced, the more critical aspect might be Work mechanical work or calories burned. If so it may also be that enhancing velocity is a good way to accomplish this.

Negative damping has been compared to methods that enhance error for rehabilitation [5, 6], though this was not our elicit goal. The reasoning is that any mistake is often made larger by negative viscosity. Error based learning leverages one of the well-known neurocomputational mechanisms of plasticity – supervised learning. This excludes the two other methods, reward-based *reinforcement* and repetitive strengthening of *Hebbian*. Error-augmentation paradigm, positive reinforcement is best suppressed so that error mechanisms can drive change in neural connections [33, 34]. Nevertheless, enhancing speed during training might be a way to simply give more experience (both errors or successes).

This study showed that negative viscosity had a strong direct effect at increasing participant speed. Participants also demonstrated improvements in movement accuracy, efficiency, and smoothness after the training. These findings are encouraging, and bolster the potential for using viscosity for clinical treatment. We are currently piloting a clinical exploration that uses negative viscosity to train chronic stroke survivors over multiple sessions. Our hypothesis is that we will see improvements in performance on standard clinical assessments, as a prelude to conducting a full clinical trial.

## Conclusions

In a direct comparison between the three force conditions that may increase movement speed, participants significantly increased their speed only as a direct effect of negative viscosity. Positive viscosity and breakthrough forces had no effect on participant speed. After the forces were removed, only negative viscosity showed significant improvements in other movement metrics measuring accuracy, efficiency, and smoothness. We conclude that training to increase movement speed should be conducted using negative viscosity. Even though we did not achieve the desired lasting effect on movement speed, the improvements in other movement parameters shows promise for negative viscosity as a potential treatment.

## Data Availability

The datasets used and/or analysed during the current study are available from the corresponding author on reasonable request.

## Abbreviations

UEFM: Upper extremity Fugl-Meyer
WMFT: Wolf motor function test
MAS: Modified Ashworth scale
